# Estimating Snow Depth and Leaf Area Index Based on UAV Digital Photogrammetry

**DOI:** 10.3390/s19051027

**Published:** 2019-02-28

**Authors:** Theodora Lendzioch, Jakub Langhammer, Michal Jenicek

**Affiliations:** Department of Physical Geography and Geoecology, Faculty of Science, Charles University, Albertov 6, 128 43 Prague, Czech Republic; theodora.lendzioch@natur.cuni.cz (T.L.); michal.jenicek@natur.cuni.cz (M.J.)

**Keywords:** UAV, forest, disturbance, snow depth, leaf area index, canopy closure

## Abstract

This study presents a novel approach in the application of Unmanned Aerial Vehicle (UAV) imaging for the conjoint assessment of the snow depth and winter leaf area index (LAI), a structural property of vegetation, affecting the snow accumulation and snowmelt. The snow depth estimation, based on a multi-temporal set of high-resolution digital surface models (DSMs) of snow-free and of snow-covered conditions, taken in a partially healthy to insect-induced Norway spruce forest and meadow coverage area within the Šumava National Park (Šumava NP) in the Czech Republic, was assessed over a winter season. The UAV-derived DSMs featured a resolution of 0.73–1.98 cm/pix. By subtracting the DSMs, the snow depth was determined and compared with manual snow probes taken at ground control point (GCP) positions, the root mean square error (RMSE) ranged between 0.08 m and 0.15 m. A comparative analysis of UAV-based snow depth with a denser network of arranged manual snow depth measurements yielded an RMSE between 0.16 m and 0.32 m. LAI assessment, crucial for correct interpretation of the snow depth distribution in forested areas, was based on downward-looking UAV images taken in the forest regime. To identify the canopy characteristics from downward-looking UAV images, the snow background was used instead of the sky fraction. Two conventional methods for the effective winter LAI retrieval, the LAI-2200 plant canopy analyzer, and digital hemispherical photography (DHP) were used as a reference. Apparent was the effect of canopy density and ground properties on the accuracy of DSMs assessment based on UAV imaging when compared to the field survey. The results of UAV-based LAI values provided estimates were comparable to values derived from the LAI-2200 plant canopy analyzer and DHP. Comparison with the conventional survey indicated that spring snow depth was overestimated, and spring LAI was underestimated by using UAV photogrammetry method. Since the snow depth and the LAI parameters are essential for snowpack studies, this combined method here will be of great value in the future to simplify snow depth and LAI assessment of snow dynamics.

## 1. Introduction

Snow is an essential component of the hydrological cycle [1]. It serves as a reliable water resource and the dynamics of snow cover play a vital function in rebalancing the global energy and water budget [2]. Due to the unique characteristics of snow, snow cover functions as an energy bank, radiation shield, insulator, reservoir, and water transport medium in the global climate-ecosystems [3]. Environmental agents interact with snow in complex ways. To predict these interactions between forest structure and snow accumulation and melting, factors such as air temperatures, incoming shortwave and longwave radiation, snow albedo, precipitation, wind speed, wind redistribution, and relative humidity, as well as snow interception by forest canopy or vegetation structure, influencing the snowpack [4], have to be determined. 

Accumulation and ablation of seasonal snowpack within forested areas exhibit very different dynamics compared to snow within open field areas [5,6]. Forests reduce shortwave radiation and increase longwave radiation due to trees as important emitters of longwave radiation. Because the decrease in shortwave radiation in forests plays a bigger role for the entire energy balance than the increase in longwave radiation, the snowmelt is much slower in forests compared to open regions [4]. 

Snow albedo that depends on the snow properties especially snow grain size is generally lower in forests than in open areas [7]. Wind redistribution, coincident with a precipitation event, can quickly alter accumulation patterns in open sites [8]. In the forests, snow storage and snow redistribution depend mainly on forest type (coniferous, deciduous) [4], although the importance of forest structure has also been highlighted as a significant factor influencing the under-canopy snow accumulation patterns and timing of snowmelt [9,10,11]. In case of frequent snowfall, snow interception increases. The differences between snow storages accumulated in open areas and in forests were reported from many world’s regions [10,12,13,14,15]. The mentioned studies reported by 30–50% lower snow storages in the coniferous forest compared to the adjacent open area depending on canopy structure. Similar differences in snow storages in forests compared to open areas have been also proved by [4] based on measurements at the same study area as the study presented in this paper. Under freezing and low-wind conditions, the intercepted snow remain in the canopies longer and enhance sublimation directly to the atmosphere. By rising air temperatures sublimation causes faster upload of intercepted snow on the forest ground intensifying the ablation rates of the snow cover [3]. Alternatively, with increasing forest cover, the interception of snow in the canopies reduces the amount of snow that accumulates on the ground, while shading reduces snow ablation compared to an open site [16]. It is certain that the effect of forest on snow accumulation and ablation acts to both intensify and reduce the energy budget of snowpack, consequently creating much greater heterogenous snow cover patterns compared to open sites [11]. 

Therefore, efforts to measure snow depth distributions at different places and different scales are crucial to simulating snow accumulation and ablation processing using, for instance, numerical models from conceptual to physically based distributed models or hydrological models that simulate snowpack quantity parameters (snow water equivalent (SWE) and snow depth), as summarized in [2]. These hydrological models are one of the common snow measurement approaches based on numerical models. The only drawback of these models is the lack of reliable meteorological observations as model inputs [17] and the complex physics of snow are still not adequately understood which hampers the development of snowpack models. Further approaches are snow pits and manual probing [18]. These traditional methods are both times consuming and delivering point-scale accuracies. To obtain basin-scale snow cover observations, conventional non-invasive remote sensing techniques have been used for monitoring snow dynamics which succeeds the potential of undersampling of point-scale snow measurements, however, are affected by detection limitations in the lower spatial and temporal resolution that characterize the measurement of snow cover [8]. Other recently existing methods include GPS-reflectometry [19,20,21] and airborne Light Detection and Ranging (LiDAR) mapping [22], Terrestrial Laser Scanner (TLS) [23,24,25,26], digital photogrammetry [27,28], tachymetry [29], Ground Penetrating Radar (GPR) [30], time-lapse photography [2,31,32], or satellite-based sensors [33]. These techniques are of high costs (e.g., airborne or helicopter flights) or only useful supplements to weather stations and manual measurements (e.g., time-lapse photography) [3]. 

Due to the miniaturization of navigation sensors, it is now possible to choose between different camera systems, spanning from heavy-weight LiDAR mapping sensors to light-weight mini-multispectral systems [16] making it possible to explore new, unforeseen research directions. Especially attractive is the low-cost technique integration of high-resolution unmanned aerial vehicle (UAV)-based digital photogrammetry, which captures small-scale spatial variabilities of snow cover. Since the development of the Structure from Motion (SfM) algorithm [34], it is possible to reconstruct georeferenced point clouds, high precision digital surface models (DSMs), and orthomosaics with a high spatial resolution, down to the centimeter level, by automatic matching of common features from a set of overlapping images [34,35]. Using the SfM method for snow cover image matching is still considered to be problematic, due to the homogeneity of the snow cover surface, but images acquired in the visual (VIS, λ = 400–700 nm) part of the electromagnetic spectrum are delivering promising results. Recent investigations of this technique of snow depth estimation used imagery taken by UAV for large to small catchments [18,36,37,38,39,40,41]. These examples have shown vertical accuracies in retrieving snow depth values from the UAV, reaching accuracies in the range of 0.1–0.3 m. This new technique provides relatively accurate solutions that may be used, not only to obtain the snow depth at high resolution in open areas, as in the studies mentioned above but can also provide information on snow accumulation and melting in forests.

The amount of forest cover is often described by estimating canopy closure (CC) or gap fraction. Also, the leaf area index (LAI) [42] is a variable to specify the forest cover by calculating the leaf area per leaf area per unit ground area. However, none of the three variables would directly represent the canopy interception through the amount of forest cover, but it can be an input variable in snow interception models [10,14,16,43]. Since UAV platforms achieve image quality equal to traditional airborne photogrammetry, downward-looking UAV imagery can be used as an indirect method for LAI estimates, which is comparable to digital hemispherical photograph (DHP) analysis of the sky and canopy [44]. The only difference is the background, which is snow-covered terrain instead of the sky [45]. Measurements and modeling, however, become more challenging with forest which is changing due to natural disturbances (e.g., bark beetle and windstorms). Although the application of UAV to measure either snow depth or LAI is not new, there is still an only limited number of published studies focusing on the use of these new sensing methods for such purpose. Linking snowpack distribution and forest LAI measurements by way of downward-looking UAV-based photogrammetry have not been done yet in forested environments. There are significant interactions between open field and forest snow processes and the impacts on snowpack evolution under forest canopy [46] which are not fully understood increasing the need for further investigations. 

The objectives of our study were to introduce a workflow for efficient UAV-based snowpack monitoring, in particular (i) the monitoring of snow accumulation and ablation processes, (ii) the assessment of canopy characteristics as the effective LAI of crowns using downward-looking UAV images (iii) to analyze the relationship between the dynamic snow depth and LAI distribution in diverse Norway spruce stand. Over a complex winter season, spanning from snow accumulation to ablation, we determined snow depth and LAI distribution in a mid-latitude montane forest small-scale environment, featuring different structure and health status, including forest-free area, healthy and disturbed forest resulting from bark beetle infestation. The UAV imaging campaigns for snow depth and LAI retrieval were accompanied with repeated manual snow depth monitoring, as well as by ground-based LAI measurements by the LAI-2200 plant canopy analyzer and DHP, used as a benchmark for testing the accuracy of our approach. 

## 2. Materials and Methods 

### 2.1. Study Test Sites

**Figure 1:**
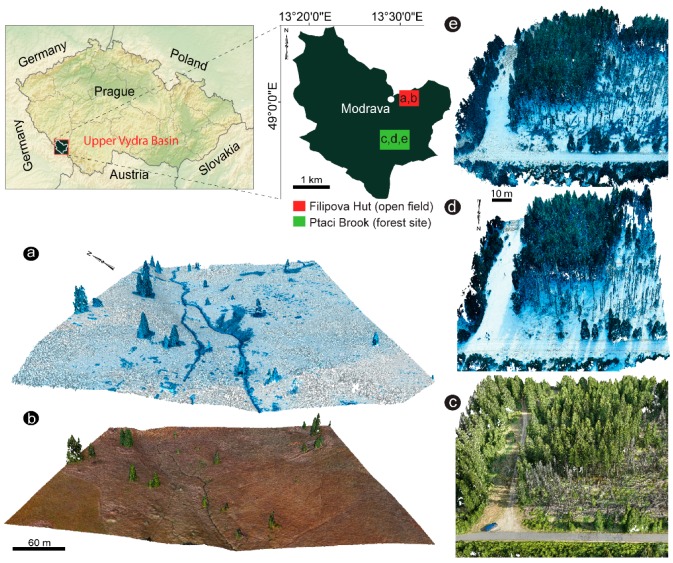
An overview map of the study area (**a**). Location of the regions surveyed with the generated 3D point cloud models. These point clouds assist in providing visual information on the snow surface and terrain type. The red box shows the open area (Filipova Hut) during snow ablation (**a**) and snow-free period (**b**), the green box displays the disturbed spruce forest zone (Ptaci Brook) during snow-free period (**c**) snow-covered (e.g., peak accumulation) (**d**), and snow ablation period (**e**)).

The study covered two different types of small scale localities within the Šumava National Park (ŠumavaNP; Bohemian Forest), Southwestern Czech Republic (Figure 1a). Data from one open area (49°1′32.67′′ N, 13°30′58.38′′ E) with meadow coverage and one forested area (48°59′8.591′′ N, 13°30′30.998′′ E) with dominant Norway Spruce (*Picea abies* (L.) Karst.), comprising a zone of healthy standing trees and an area of profoundly disturbed forest, were utilized for this study (Figure 1b).

These areas are located in a mid-latitude montane area with altitudes of 1070–1150 m a.s.l., featuring mild topography. Granite intrusions and metamorphic rocks shape the geology of the whole area. Soils are rather shallow with high permeability (cambisol, podzol) with a significant portion of hydromorphic and organic soils. Mean annual air temperature at Churanov station is 5 °C (located at 1118 m a.s.l. 8–12 km from study sites) with mean seasonal temperature −3.1 °C from December to February. Mean annual precipitation reaches 1228 mm (climate station is located near the open study site). The study area has snow-dominating runoff regime with the highest runoff volume during spring season caused by melting snow. Seasonal runoff from March to May reaches 41% of total annual runoff in the Ptaci Brook (data period 1980–2014). 

The study area has experienced significant changes in land use and canopy structure. The mountains were covered by a virgin forest until the 18th century when it was replaced by a Norway spruce monoculture for the wood industry. Introduction of the Norway spruce instead of the natural species required large-scale artificial drainage that affected the natural hydrological regime of the area. Since then, the region is repeatedly affected by bark beetle outbreaks. The recent large-scale bark beetle infestation is occurring in consequence of windstorms in Bavarian National Park in 1984 that started the bark beetle outbreak and were heavily accelerated since Kyrill windstorm in 2007. In result, the extensive forest disturbance is consequently reaching the boundary part of the mountain range. As the study sites are located in the National Park, the disturbed forest is left to the natural processes. Hence, all stages of the forest disturbance, decay and regeneration can be identified in the study area and affect the processes of snow accumulation and ablation.

The open area (~0.04 km^2^) was characterized by medium-high grass and isolated tree vegetation (Figure 1c,d). A small meandering creek cut the topography. Along the river-creek, some peaty meadow vegetation occurred. Slopes were NW-SE and NE-SW oriented. Snow conditions were undisturbed. The open area was accessible during both the winter and summer seasons. Usually, only moderate to low snow depth variability could be expected here due to meadow coverage and its year-round exposure to the sun and wind.

The forest area (~0.012 km^2^), which has been affected by bark beetle (*Ips typographus* L.) outbreaks since 1990 [47], reveals large canopy gaps due to the complete collapse of the tree layer (Figure 1e–g). The effects of windstorms and bark beetles were a natural part of the dynamics of the spruce forest. Therefore, dynamism in the vegetation is still evident. In 2017, even more, dead trees littered the ground than in 2016, especially in the upper part of the study plot. The remaining healthy forest canopy was around 10–25 m tall. Consequently, the forest area could be divided into two parts: an upper section with fallen branches and rotten wood left on the stand, and a lower section with a healthy spruce forest containing dead trunks standing among the trees. Lower snow depth variabilities were expected here because the area was not exposed to high winds due to the surrounding trees, which protect against the wind and, in part, the sun. Even so, within-plot snow depth, interception, and snowmelt distributions should be expected to be higher in the forest than in an open area, because of standing and lying dead trees. The forest area was conveniently accessible by car during summer but less during winter time due to prepared cross-country ski-tracks. Peak winter accumulations at both study sites were interrupted by two snowmelt events caused by rain-on-snow conditions and high snow densities.

### 2.2. UAV Monitoring

UAV surveys consisted of two snow-free and three snow-covered imaging campaigns, completed by additional ground measurements of snow depth that were performed in the spring part of the 2017 winter season, covering a phase with the direct consequence of snow accumulation and ablation. Snow-covered images were obtained during the period of snow accumulation on the 3 March 2017; and during snowmelt on the 15 March 2017, at the forest site, and on the 24 March 2017 at the open field area. Snow-free images were taken directly after snowmelt on the 30th of April 2017 at both sites when snow was already absent, and the ground vegetation was still flattened from the snow cover. 

**Figure 2:**
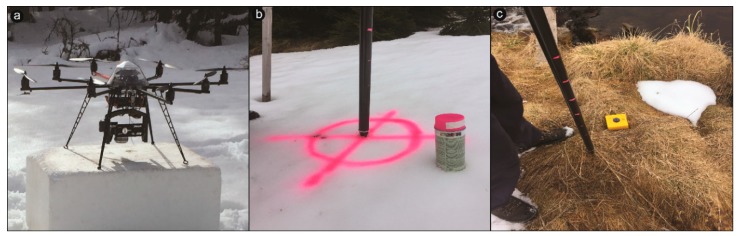
(**a**) Applied ARF MicroKopter Okto XL equipped with RGB Lumix-GX7 camera (Panasonic Corporation, Osaka, Japan); (**b**) Marked ground control points (GCPs) and checkpoints (CPs) with visible pink spray during all winter campaigns; (**c**) Yellow geodetic pits and white papers served as GCPs during the snow-free campaigns.

The UAV platform, the MicroKopter ARF-Okto XL (HiSystems GmbH, Moormerland, Germany, Figure 2a), was used for imagery acquisition of snow depth distribution and LAI retrieval. This octocopter is suitable for performing photogrammetric flights over limited areas (<1.5 km^2^). It was equipped with a commercial RGB camera: the Panasonic Lumix DMX-GX7 featuring a 16 MP Micro Four Thirds (17.3 × 13 mm) CMOS sensor fitted with a Panasonic 20 mm f/1.7 prime lens. To achieve a very high spatial resolution of centimeter-scale ground sampling distance (GSD), the flight altitude varied between 46 and approximately 73 m. Images were taken each second, with a 400 ISO speed range. The manual focus was set to infinity, to avoid focusing on single, undesired objects. All images were captured in the VIS (400–700 nm) part of the electromagnetic spectrum. 

We programmed the flight path with waypoints for taking autonomous images including flight height and flight time. It was set in the corresponding MikroKopter flight planning tablet tool (MK-TT) and transferred to the copter. The typical flight duration was around 20 min in the summertime, while in the wintertime with temperatures below −10 °C, the flights were much shorter due to the rapid drain of the Lithium Polymer (LiPO) batteries. To achieve seamless coverage of the study sites, and to get a sufficient number of point pairs in the imagery, the front and side lap were both set to 80%. Before a survey flight, well distributed, predefined ground control points (GCPs) were placed over the study sites to secure accurate georeferencing of the imagery (Figure 2b,c). Some of the GCPs served as checkpoints (CPs) to assess the accuracy of the model. Permanent GCPs were marked with fixed wooden sticks to secure the same collection of GCPs during snow cover, but most of them disappeared. The remaining rods served then as permanent markers and were completed by temporary GCPs, either bullseyes or colored pink crosses, sprayed over the snow cover (Figure 2b). 

For accurate positioning of GCPs and additional snow depth samples, the Global Navigation Satellite System (GNSS) Topcon HiPer SR was used in Real Time Kinematics (RTK) mode. This device measures the coordinates of the center of the GCPs with a horizontal precision of 0.01 m to 0.03 m and vertical of 0.02 m to 0.05 m at each point. To keep this level of accuracy, for each GCP and CP measurement the online RTK corrections were used as recommended by the Czech State Administration of Land Surveying and Cadastre Authority. For RTK corrections, a Virtual Reference Station (VRS) was used, which was located 5 km from the area of interest. The VRS is calculated based on real reference stations among the national permanent GNSS reference stations. Detailed technical specifications of both UAV and the camera system are included in Table 1.

### 2.3. Photogrammetric Processing

Before processing the images, manual pre-selection was done to remove blurred photos. All suitable images were then processed using Agisoft PhotoScan Pro (version 1.2.6) [48] to reconstruct snow-free and snow-covered DSMs, together with maps of snow depth distribution and orthomosaics from both study sites. PhotoScan is commercial software that enables full control over the all steps of photogrammetric processing, ranging from image alignment and sparse- and dense-point cloud generation, to the building of the DSM and orthoimagery [49,50]. During the processing in Agisoft PhotoScan Pro, the imagery was aligned choosing “accuracy-medium”, key point limit 40,000 and tie point limit 20,000. The point clouds were generated by using the settings “quality-medium” and “depth filtering—moderate”. After the initial image alignment process and removing evident outliers, the sparse clouds were manually georeferenced by identifying the GCP in the matched pictures and assigning them the coordinates from the GNSS post-processing (S-JTSK Krovak East North (EPSG 5514)). The checked markers which were GCPs were applied for the optimization procedure, whereas the chosen unmarked markers, in our case the CPs, served as control points to quantify the accuracy of the results and thus the total RMSE. For the absolute orientation, eight selected GCPs for the snow-free campaign were used, while six GCPs served as CPs to evaluate the accuracy of the alignments at the open site (Figure 5a). Nine GCPs were distributed with six GCPs acting as CPs for the snow-covered DSM processing at the open site (Figure 5b). Five GCPs were used for the snow-free image campaigns with four CPs in the forest zone (Figure 6a). For the other two campaigns of snow-covered imagery, four to five GCPs with four to five CPs were derived (Figure 6a,b). For the dense cloud reconstruction process “medium reconstruction quality” in Agisoft PhotoScan Pro was used to balance the computational costs with the required level of detail. The raw point clouds were exported, with a spatial resolution of 0.10 m, to perform further point cloud editing and classification in CloudCompare software [51]. Ground points were extracted from the point cloud by removing trees based on the Cloth Simulation Filter (CSF) [52]. To remove light to hard shadows on snow-covered point clouds, we used a k-means clustering algorithm after [53]. This algorithm classified the point clouds based on their RGB attributes with a Nearest Distance type classifier. The clusters were created on a training subset and then applied for the whole dataset. After classification, segmentation, and shadow removal, DSMs of 0.10 m resolution were built in GIS software [54]. Co-georeferencing were applied to georeferencing both snow and bare soil DSMs based on common GCPs used for both snow-free and snow-covered DSMs [41]. 

### 2.4. Snow Depth Data Acquisition and Analysis

For each of the studied areas, during snow-free and snow-covered period GCPs were measured. During winter manual point measurements of snow depth (H_GCP_) were sampled precisely above the measured GCP positions so that the x, y and z positions of the GCPs and the positions of the corresponding snow probes could be measured simultaneously. Additional arranged snow depth samples (H_T_) were taken at both sites to improve the overall accuracy of the generated surveys. These H_T_ values were estimated from the means of five individual snow depth measurements taken in a 1m radius. A total of 105 H_T_ samples in a plot of 170 × 125 m at the open site and 36 H_T_ samples in a rectangle plot of 50 × 50 m at the forested zone were collected for each measurement survey. The idea was to cross-check the results of these two data sets against each other to ascertain if an increased quantity of ground measurements would significantly impact the overall precision of the survey when comparing them with the estimated (H_UAV_) snow depths. Additionally, to reduce the total error by obtaining the average of several snow depth readings at the point of observation. It must be noted here that the additional H_T_ samples at the forest site are conducted every year due to long-term snow-cover monitoring [4,55,56]. Therefore, the study area at the forest zone was only partly covered by snow depth probing. These samples, however, were enough for the quality check, and they were also used during snow accumulation and snow ablation for the computational interpolation of manual snow depth estimates with the winter LAI obtained by the three methods. We used both datasets of manual snow depth measurement (H_GCP_ and H_T_), one with more and one with fewer ground measurement reference points, to implement a preliminary evaluation of UAV performance in retrieving point values of snow depth from the DSMs of difference (DoD). 

### 2.5. LAI Assessment

**Figure 3:**
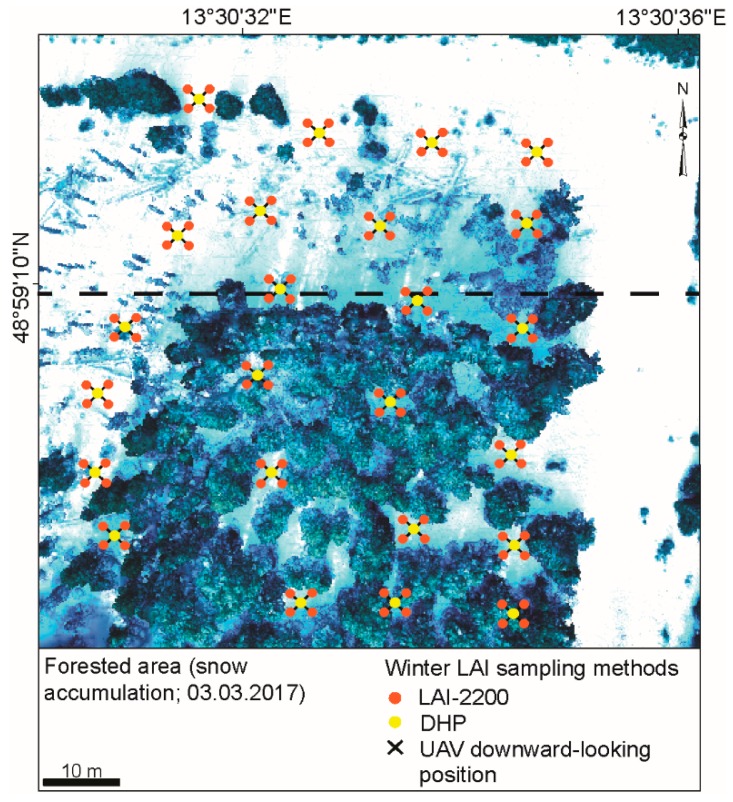
The position of all leaf area index (LAI) field surveys are displaying 24 points. Green points present the digital hemispherical photography (DHP) position. Orange points correspond to the LAI-2200 canopy analyzer measurements as the average of four individual LAI values measured in a cross shape with 1 m distance from the cross arm ends to the center. The blue cross signs show the position of downward-looking images as seen from the UAV performance by the camera used for the LAI retrieval. Black dashed line indicates the border between healthy and bark beetle-damaged forest stand.

LAI measurements (Figure 3) were performed respectively at the 3 and 24 March 2017. To test the reliability of the UAV-based LAI flyovers, additional ground-based measurements using the Li-Cor LAI-2200 plant canopy analyzer (Li-COR Inc., Lincoln, USA) [57] and DHP, were conducted. During the first campaign, a little snow was still present at a few tree crowns. Additionally, dead lying wood on the ground was already snow-free. These optical methods are for indirect field measurements of LAI, producing the so-called effective LAI index (LAI_eff_; [58]). The optical detector of the LAI-2200 sensor is composed of five different detectors in concentric rings, each of which views a portion of the hemisphere, such as 0–13° (1st ring), 16–28° (2nd ring), 32–43° (3rd ring) and 61–74° (4th ring) [59]. This allows for the computation of radiation transmission caused by the canopy in the blue part of the spectrum and therefore calculates the LAI by comparing differential light measurements above and below the canopy [60]. Above measures served as a reference and were taken before each set of below-canopy sampling outside the forest. Below-canopy measurements with the LAI-2200 were made about 1 m above the ground in a grid of 50 × 50 m, at 10 m intervals, for 24 points at the forest site. 

Each plot was estimated by means of four individual LAI values measured in a cross shape with ~1 m distance from the cross center, similar to the manner employed in [61]. The center point of the cross of each plot was marked with a blue cross symbol, which could be detected from the UAV platform to verify the accuracy of it. All readings were carried out in the morning. At both dates, the weather conditions varied between partly clear sky and overcast conditions. A narrower view cap of 270° was used to occlude the influences of the direct sun and the operator in the measurements [57]. Post processing was conducted with the FV2000 software (Li-COR Inc., Lincoln, NE, USA) [57]. To achieve LAI_eff_ results closer to the true LAI, while maintaining consistency in comparison with the LAI_eff_ produced by DHP, the fifth ring was excluded during data processing [62]. Hence, the view of the zenith angles corresponds to 58° which is approximately equal to the ring with the largest view angle of the hemispherical camera used in this study.

The camera was mounted on a tripod at a fixed height of 1 m above the ground. The lens aperture was fixed, and the exposure time was set to auto-mode. The geographic orientation and circular extent of the hemispherical image were applied in a northerly direction, which corresponds to 0°. Similar to the LAI-2200 plant canopy analyzer, the DHP computes the gap fraction through the camera with a hemispherical lens (fisheye) [63]. In total, 24 points were measured. LAI was processed using the Gap Light Analyzer (GLA) software (Cary Institute of Ecosystem Studies, New York, USA) [64,65].

**Figure 4:**
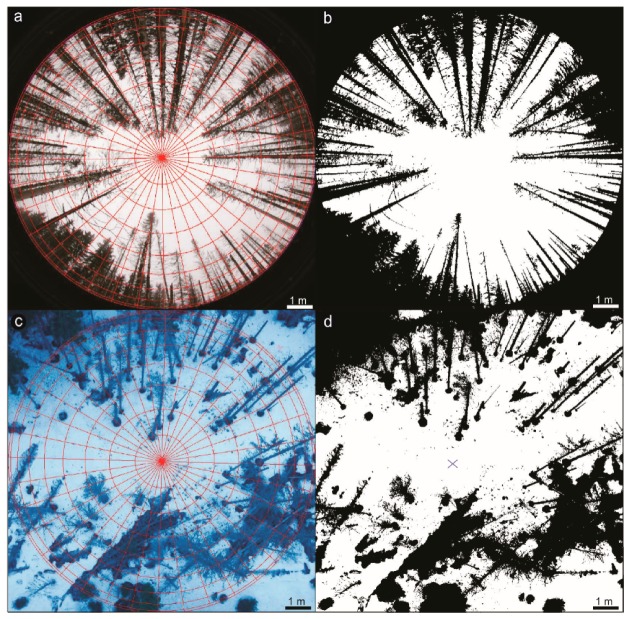
Illustration of Gap Light Analyzer (GLA) environment for a fisheye lens. (**a**) Registration of image—setting the reference of north orientation and setting the 180° range; (**b**) Settings of the called threshold value—the hardness of determination which pixels would be computed like “clear sky” and which would be computed as “view obstacles”; (**c**) Illustration of GLA environment for rectangle lens with orthographic projection settings. Registration of image remains the same like for the fisheye lens, as well the thresholding of the image in the LAI ground measurement plot of (**d**) showing the blue cross sign as seen from the UAV downward-looking images.

The UAV surveys for LAI retrieval were performed in partly sunny to overcast conditions. Overcast conditions are more preferable to avoid shadows on the forest floor [60], which can hamper exact LAI retrieval, but images could be analyzed with adequate accuracy. The UAV-based LAI_eff_ values were estimated from measurements that were taken at two different altitudes (at ~50 m and ~65 m) to determine whether there were observed differences in the LAI_eff_ retrieval between the optimal height and higher ones. The downward-looking UAV images were taken from nadir position with a field of view (FOV) of 48°. To catch approximately the same positions of ground measurements, images were obtained at an interval of one second. Altogether, 24 points were measured within the Norway spruce stand and marked by a blue cross sprayed on the snow-cover, like those recorded by the other two optical methods. All LAI values measured in this study are effective values, so the clumping of the needles was not taken into account. GLA software, used for LAI assessment by DHP (Figure 4a,b), was also applied for LAI determination, based on UAV downward-looking images (Figure 4c,d). This allows for the processing of both custom fisheye lens distortions, and also of standard lens projections [64,65]. Here, instead of a polar projection, which is used for fisheye lenses, an orthographic standard projection for rectangle images was used in GLA software. GLA computes all zenith angles from 0 to 60° for its effective LAI. The manual segmentation based on classifying pixels as either canopy or snow. The environment of the GLA is shown in Figure 3, and the input parameters are listed in Table 2.

## 3. Results

### 3.1. Study Sites and Data Acquisition

At the open site, the total of 2093 images was acquired on two different dates from March (during snow ablation) to the end of April (after snowmelt). The sum of two DSMs and orthoimages of ~0.10 m spatial resolution was generated from the field surveys. The absolute accuracy of the derived DSMs (snow-free and snow-covered maps), relative to the measured surface points are summarized in Table 3.

At the forest site, featuring different health status of the forest stand, a total of 2494 images were captured in three campaigns from March (during snow accumulation and snow ablation) to the end of April (after snowmelt) with the same data acquisition parameters as at the open site. The total of three DSMs and orthoimages of ~0.10 m spatial resolution was generated and the absolute accuracy of the derived DSMs (snow-free and snow-covered) of the root mean square (RMSE) for x, y, and z together with the area total error in m of the CPs are listed Table 3.

### 3.2. UAV-Based Snow Depth Estimations vs. Manual Snow Depth

**Figure 5:**
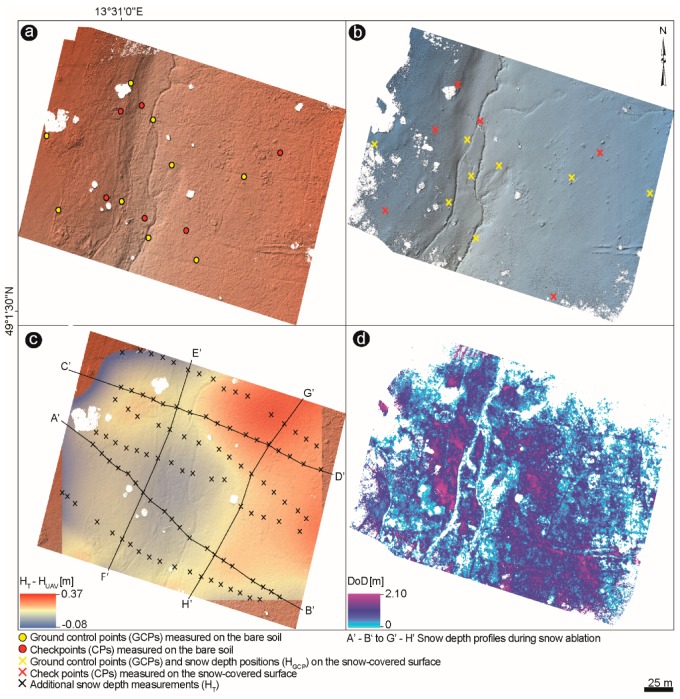
(**a**,**b**) Hillshaded snow-free and snow-covered DSMs and detail surface visualization of the open site (Filipova Hut) with the applied ground control points (GCPs) and checkpoints (CPs). GCPs taken during snow cover served as well as reference snow depth points at the GCP positions (H_GCP_; pink crosses). Overlapping snow-free and snow-covered GCPs were selected in support of co-georeferencing the snow-free DSM with the snow-covered DSM. (**c**) Map of interpolated spatial distribution of the snow height differences between the supplementary H_T_ probes and estimated H_UAV_ measurements underlying the bare soil hillshade (**d**) DSM of difference (DoD), produced by subtracting the snow-covered DSM from the snow-free DSM.

**Figure 6:**
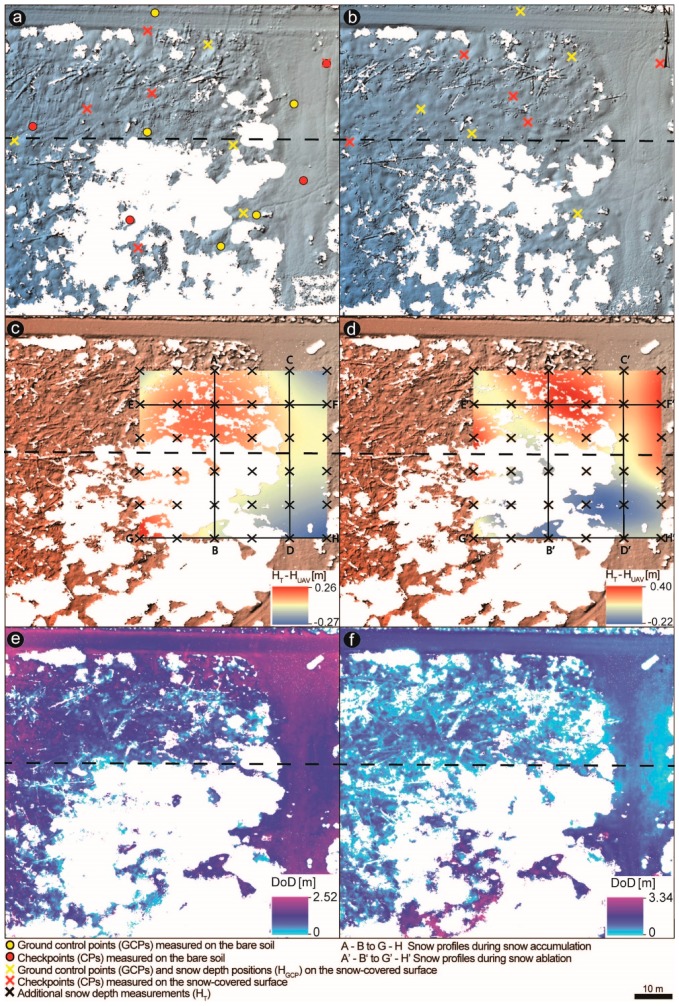
(**a**,**b**) Hillshaded snow-covered DSMs during snow accumulation and ablation, and detail surface visualization of the forest site (Ptaci Brook) with the applied ground control points (GCPs) and checkpoints (CPs). GCPs taken during snow cover served as well as reference snow depth points at the GCP positions (H_GCP_; pink crosses). Overlapping snow-off and snow-on GCPs were selected in support of co-georeferencing the snow-free DSM with the snow-covered DSMs. (**c**,**d**) Maps of the interpolated spatial distribution of the snow height differences between the supplementary H_T_ probes and estimated H_UAV_ measurements underlying the bare soil hillshade. (**e**,**f**) DSMs of difference (DoDs).

A total of five DSMs were generated from the winter during snow accumulation and ablation, and from the snow-free fieldwork campaigns. The hillshades computed from UAV data during snow-free and snow-covered time, along with the applied GCPs, are shown in Figure 5a,b, for the open field. For the forest zone, only the hillshades during peak accumulation and ablation are visualized (Figure 6a,b) with the corresponding GCPs. GCPs, taken during wintertime also served as reference snow depth points at the GCP positions (H_GCP_) and were selected in support of co-georeferencing the snow-free DSMs with the snow-covered DSMs.

The difference between the supplementary H_T_ probes and estimated H_UAV_ measurements are visualized as interpolated maps (Figure 5c and Figure 6c,d ), showing the spatial distribution of the snow height differences. Three DoDs were created (Figure 5d and Figure 6e,f ) based on standard photogrammetric procedure Usually, by subtracting the snow-free DSMs from the snow-covered DSMs, the snow depth values appeared negative in the maps, which were classified as outliers but not masked out, and depicted as zero values in the maps, for readability. This discrepancy has already been noted during photogrammetric surveys [27,28,40] and can be attributed to the effect of compressible vegetation and instrumental precision [28,66].

The snow height differences were ascertained as mean values within 1 m buffer to be comparable with the H_T_ samples determined from the mean of five snow depth measurements taken in 1 m radius. White gaps of the created DSMs mark the filtered vegetation (e.g., trees and bushes) and might be due to shadows or poor matching over some area of the snow surface (any resulting couple points in the horizontal plane due to the homogeneity of snow). Kernel Interpolation was used to display the spatial distribution of the snow height differences between H_T_ probes and estimated H_UAV_ measurements (Figure 5c and Figure 6c,d ). The Kernel Interpolation allows the prediction of the output surface with smoother barriers [67] and uses the shortest distance between points so that points on the sides of the specified nontransparent (absolute) barrier are connected by a series of straight lines. It is advantageous because it leaves the parts out which are not snow-covered, or where trees were removed from the point cloud.

The snow height differences were ascertained as mean values within 1 m buffer to be comparable with the H_T_ samples estimated from the mean of five snow depth measurements taken in 1 m radius (Figure 6). White gaps of the created DSMs display the filtered trees and dead lying trunks on the ground and might be due to shadows or poor matching over some area of the snow surface (any resulting couple points in the horizontal plane due to the homogeneity of snow). The snow depth validation outcomes are summarized in Table 4. Statistics include the mean bias, the correspondent SD, the mean absolute error (MAE), and the RMSE between H_GCP_ and H_UAV_, as well as H_T_ and H_UAV_. Mean bias quantifies the mean magnitude of the over- (positive values) or under- (negative values) prediction of the DSM concerning manual snow depth measurements. The SD quantifies the variability of the error. Both MAE and RMSE determine the accuracy between the manual snow depth observations (H_GCP_ and H_T_) and the estimated UAV-based snow depth values extracted from the DoDs in relevant to H_GCP_ points and H_T_ area. The results indicate an overall average performance for all the surveyed areas, with results agreeing with relevant literature. For instance, [37] reached an accuracy of the DSMs relative to the measured variable surface points due to dynamic conditions with varying RMSE from 0.04 m to 0.19 m with few problematic flights showed larger RMSE of up to 0.32 m [38] estimated snow depth distribution with an RMSE of 0.30 m. Better results below 0.30 m were predicted by [41] with RMSE between 0.05 m and 0.18 m. 

**Figure 7:**
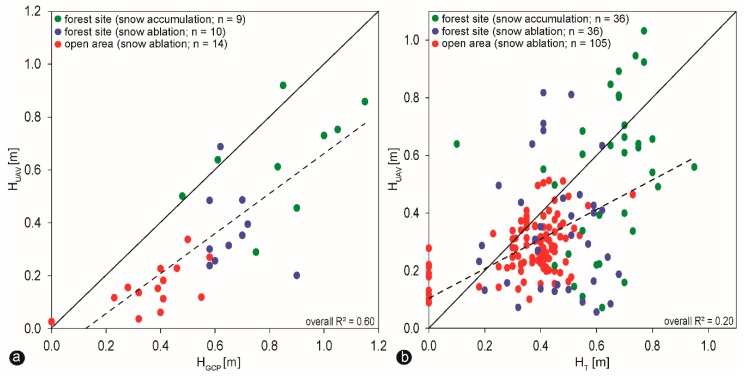
(**a**) Snow depth measured by UAV (H_UAV_) compared to the reference snow depth measured at GCP positions (H_GCP_) at all study sites. **(b)** Snow depth measured by UAV (H_UAV_) compared to the reference snow depth measured in a denser network (H_T_) at all study sites. The dataset in (**b**) features a significantly higher snow depth variability (R^2^ = 0.20) than dataset formed by snow depth measurements taken at GCP positions (R^2^ = 0.60). Graphs show the regression functions (dashed lines) and 1:1 correspondence (solid lines).

**Figure 8:**
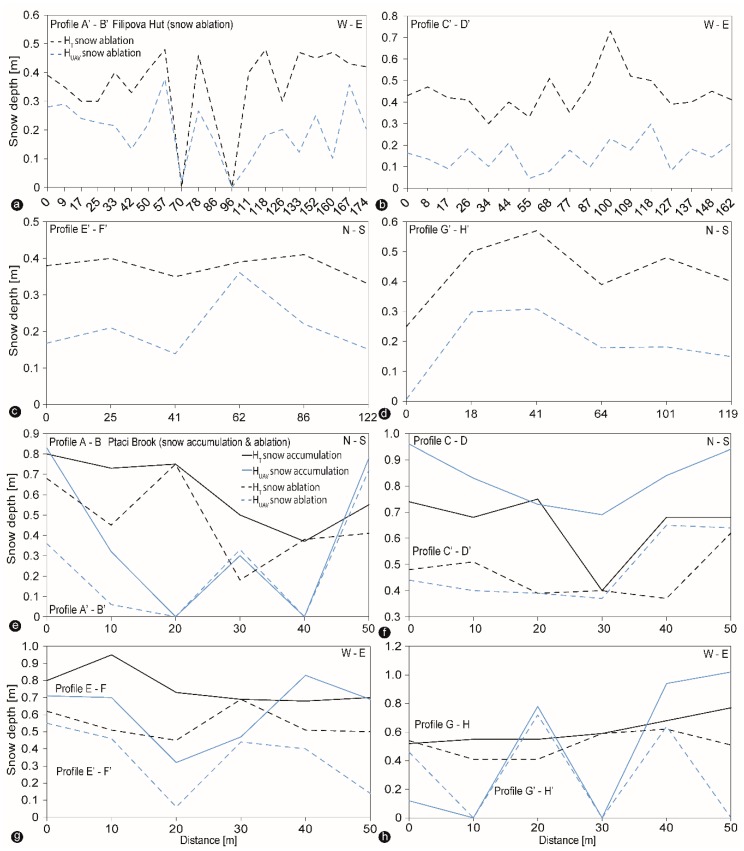
(**a**–**d**) Snow depth profiles between estimated H_UAV_ measurements and manual probing H_T_ across the open area in West-East and North-South direction during snow ablation (profiles A’-B’ to G’-H’). (**e**–**h**) Snow depth profiles between estimated H_UAV_ measurements and manual probing H_T_ across the forest site in West-East and North-South direction during snow accumulation (profiles A-B to G-H) and ablation (profiles A’-B’ to G’-H’).

One-to-one (1:1) comparisons between all snow depth measurements (H_GCP_ and H_T_) and estimated H_UAV_ snow values are provided in Figure 7a,b. The error does not change sufficiently with the snow depth (Figure 7a), showing a moderate overall correlation by all values taken at GCP positions, whereas the overall correlation coefficient is considerably weaker when comparing HT measurements with H_UAV_ estimated snow depth values (Figure 7b). Most of the H_UAV_ snow depth values are underestimated, especially in the open area (Figure 7b). The similar slope of regression line both in Figure 7a,b suggest the relative good robustness of result when using fewer measurement points. However, the much more significant variance in Figure 7b shows that only 10 GCPs cannot capture the variability of snow depth in such complex terrain (many small depressions and elevations due to windthrow, hampered the results). The decision to use a denser grid of arranged ground measurements increased the spatial coverage, snow depth variability and therefore much more confident. In addition to the above consideration, we extracted snow depth profiles of the estimated H_UAV_ measurements from the DoDs (Figure 5d and Figure 6e,f ) and plotted them along with the manual taken H_T_ snow depths (Figure 5c and Figure 6c,d ). Figure 8 shows the produced transects in respectively West-East and North-South directions (four profiles for each snow event in total) during snow ablation at the open site and snow accumulation and ablation at the forest site. 

Comparison of the estimated H_UAV_ snow depth and manual probing H_T_ in Figure 8 reveals that the H_UAV_ measurements run a relatively similar course like manual probing but by nearly two orders of magnitude lower, especially in the case of the open area (Figure 8a–d). Systematically superposition of manual probe locations is observed compared to the lower corresponded run of the estimated H_UAV_ snow depth profile (Figure 8a–d). Except one H_UAV_ point is higher than H_T_ as shown in Figure 8a due to different underlying vegetation structure. The forest transects (Figure 8e–h) show more variability between the H_UAV_ snow depths and H_T_ data along the profiles. These transects show partly the H_UAV_ snow depths reproducing all of the features revealed by the manual probes, despite the several confounding effects (e.g., tree canopy, tree stumps, and dead lying wood on the ground or changing vegetation structure). In few locations along the profiles, the H_UAV_ values indicate changes up to 0.5 m lower than revealed by the probes within the healthy forest part (e.g., Figure 8e) during both snow conditions displaying zero distributions. Higher appearing H_UAV_ values than H_T_ can be explained by snow-covered wood features, such as lying dead trunks or dead wood on the ground or due to different underlying vegetation structure, especially at the forest site (Figure 8a,e,f,g,h).

### 3.3. Optical Indirect Estimation of LAI_eff_

**Figure 9:**
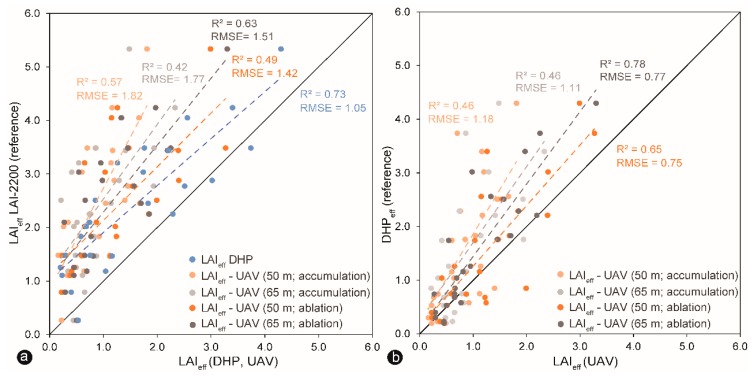
Comparison of effective LAI values produced by LAI-2200 as a reference (plot **a**; y-axis); hemispherical digital photographs (DHP) as a reference (plot **b**; y-axis), and UAV (x-axis in both plots) processed in FV2000 and Gap Light Analyzer (GLA) software. Graphs show the regression functions (dashed lines) and 1:1 correspondence (solid lines).

By taking LAI-2200 canopy analyzer and DHP as benchmarks, the correlations between the measured values of the UAV-based LAI_eff_ and the reference values were calculated and plotted in Figure 9a,b. 1:1 relationship across these methods were significant in a few instances. A high relationship was obtained between the two reference methods DHP and LAI-2200 (Figure 9a). The correlations between the UAV-based LAI_eff_ data and LAI-2200 were less strong (Figure 9a). The correlations between UAV-based LAI_eff_ observations and DHP were as good as between both reference methods (Figure 9b). Most of the observed values were all distributed above the 1:1 line (Figure 9a,b). The observed LAI values using UAV and DHP methods were underestimated (Figure 8a). A similar 1:1 relationship was shown in the scatter plot in Figure 9b. The observed UAV values were almost all above the 1:1 line except some values between 0 and 3. In both cases, the observations provided overestimations. RMSE indicates the accuracy of all utilized methods; Figure 9a shows the UAV-based LAI estimation has a lower accuracy comparing to the observed DHP values with LAI-2200 observations. On the contrary, Figure 9b shows a higher accuracy by comparing the UAV and DHP methods.

### 3.4. Relationship between Indirect Winter LAI_eff_ and Snow Depth

**Figure 10:**
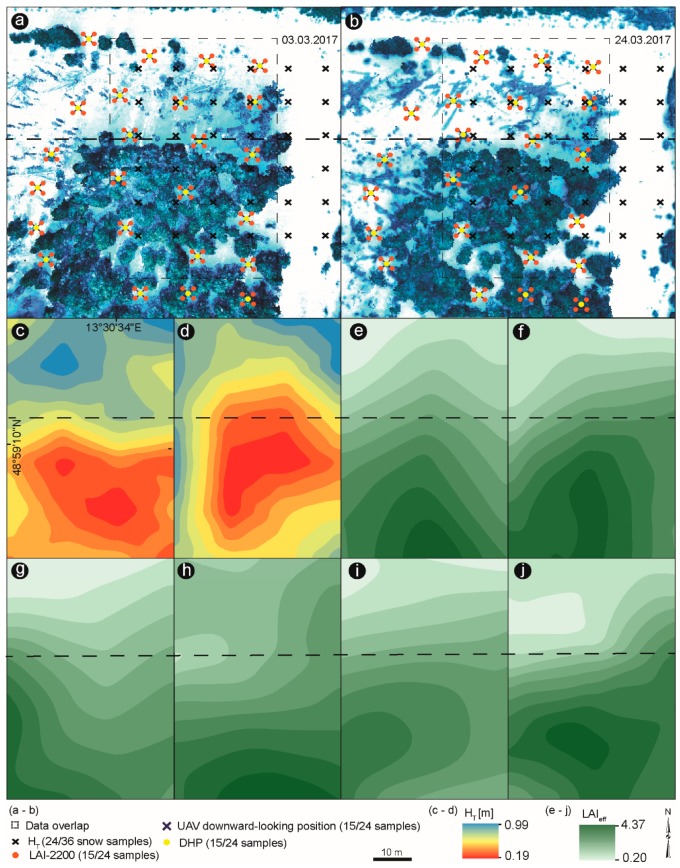
Field plot sampling design. (**a**,**b**) Snow depth measurements (H_T_) taken at 10 m interval during snow accumulation and snow ablation period with visualized ground-based measurements of LAI and UAV downward-looking positions at 24 points. Interpolated H_T_ probes during snow accumulation (**c**) and ablation (**d**). Interpolated ground measurements of LAI-2200 (**e**); digital hemispherical photography (DHP) (**f**); UAV-based LAI_eff_ at 50 m (**g**) and 65 m (**h**) during snow accumulation; UAV-based LAI_eff_ at 50 m (**i**) and 65 m (**j**) during snow ablation. All data were interpolated within the data overlap frame, which displays the overlapping area of snow depth sampling (H_T_) and ground-based measurements of LAI. Different colors indicate different values of snow thickness and LAI values. Black dashed line separates the healthy forest from the bark beetle-infected forest stand.

To provide better visualization of the H_T_ measurements and LAI_eff_ values taken by the different sampling methods, these data points were also interpolated using Kernel Interpolation. All data presented in Figure 10a,b were considered for data interpolation. However, only the overlapping data points within the figures were valid. The interpolated H_T_ values during both snow conditions are shown in Figure 10c,d, whereas the interpolated LAI data are presented in Figure 10e,f. During snow accumulation, H_T_ ranged between 0.38 m and 0.99 m (Figure 10c), while during snow ablation the H_T_ measurements varied between 0.19 m and 0.68 m (Figure 10d). In both cases, higher snow depth values appeared in the upper part of the study plot where the forest was mostly damaged, and less vegetation canopy was existent. On the contrary, the lower part, which mainly consisted of healthy trees, was below the tree canopy and therefore, lower snow depth was present. This trend goes hand-in-hand with the ground-measured LAI and UAV-based LAI estimates that show an opposite pattern (Figure 10e–j). The higher the LAI values, the lower the snow depth, and vice versa. The apparent north-south trend how visible in Figure 10c–j is not affected by the applied interpolation technique, but it reflects the captured data in the field and thus makes it clear how canopy structure plays a primary control on snow depth. 

**Figure 11:**
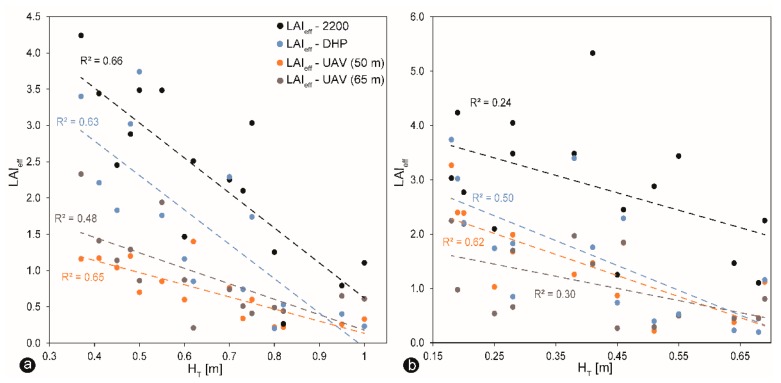
The plotted relation between effective LAI (LAI_eff_) estimates and manual snow depth samplings (H_T_) during snow accumulation (**a**) and during snow ablation (**b**) at the zone of overlapping ground-based measurements. Dashed lines represent linear regressions.

The LAI-2200 plant canopy analyzer measured the highest LAI values, with maximum LAI estimates of 5.34 in the denser part of the forest, while the UAV-based LAI measurements showed the lowest maximum values of 3.27 and 3.30, taken at 50 m and 65 m, respectively. Overall, all methods showed the same pattern of LAI estimates (Figure 10e–j), with higher LAI values indicating lower snow depth. It is well accepted that snow storages in the coniferous forest characterized by high LAI values are lower compared to open environments, or as in this case, damaged portions, because of the processes of interception, sublimation, evaporation and wind redistribution [5,10,15,67]. Consequently, there is also a statistically significant correlation between LAI_eff_ and manual H_T_, particularly during snow accumulation (Figure 11a). Considering that the relationship was developed from ground-measurements and by UAV at two different altitudes, the fit is entirely fair (Figure 11a). The R^2^ between H_T_ and the LAI-2200 plant canopy analyzer was the highest during snow accumulation (Figure 11a). LAI_eff_-UAV (50 m) and DHP showed similar correspondence. The lowest relationship was between LAI_eff_-UAV (65 m) and the LAI-2200 plant canopy analyzer. The influence of winter LAI can be seen in its control of the snow depth within the different forest stands during snow accumulation and ablation. LAI_eff_-UAV (50 m) and DHP presented the best fit during snowmelt, whereas LAI_eff_-2200 and LAI_eff_-UAV (65 m) exhibited an instead scattered relationship (Figure 11b).

## 4. Discussion

### 4.1. Snow Depth Validation of Open vs. Forest Site

Snow depth variabilities were captured over the meadow and complex underlying topography in the forest. High snow accumulation zones were linked with topographical depressions, especially at the open area, whereas only some parts were linked more efficiently at the forest site during both snow events. However, as with any remote sensing technique, errors can exist at any stage of image acquisition or analysis. The comparison between measured and estimated snow depth for the H_GCPs_ probes ranged between 0.08 m for the open area through 0.15 m during spring snow accumulation in the forest and 0.09 m during spring snow ablation within the forest stand. The snow depth estimates compared between a denser network of snow probes varied between 0.16 m for the open field through 0.32 m during snow accumulation to 0.31 m during snow ablation for the H_T_ measurements at the forest site. With an increasing number of sample points, the results deteriorated, especially at the forest site. Notably, it makes a difference between randomly selected fewer snow probes (H_GCP_) taken within canopy gaps or near the canopy edges than a denser network of snow probes (H_T_) taken as well under canopy positions. In the forest a denser network of snow probes is more sensible to sources in the context of uncertainty, it highlights the complexity of the terrain and vegetation-induced variability in snow properties. However, the results confirmed that the overall robustness of our model capturing snow depth variabilities over more sample points does not differ much at the open site. A similar finding is supported by [18] that a higher number of manual snow probes do not necessarily affect the overall precision of the survey regarding the RMSE as well.

Likewise, the accuracy of the precision didn’t improve by taking more ground samples in this case. However, these statistics are based on a more limited validation power (point-scale measurements) than using other methods, e.g., Airborne laser scanning (ALS). ALS is capable of accurately mapping snow depth for small to large aerial extents, and at high spatial resolution accuracy of snow depth estimates of 0.15 m as reported by [22]. Additionally, using terrestrial laser scanning (TLS) might be more efficient to improve ground mapping in the forest environment. Data acquisition with TLS is time and manpower consuming, and only possible at easily accessible spots [38]. This study proves that snow depth statistics are consistent with previous studies [18,41,64,68,69,70,71]. It also confirms that the UAV application can reach high-quality snow depth estimates in different environments and vegetation, although lower errors were expected at the open area compared to the forest site. 

**Figure 12:**
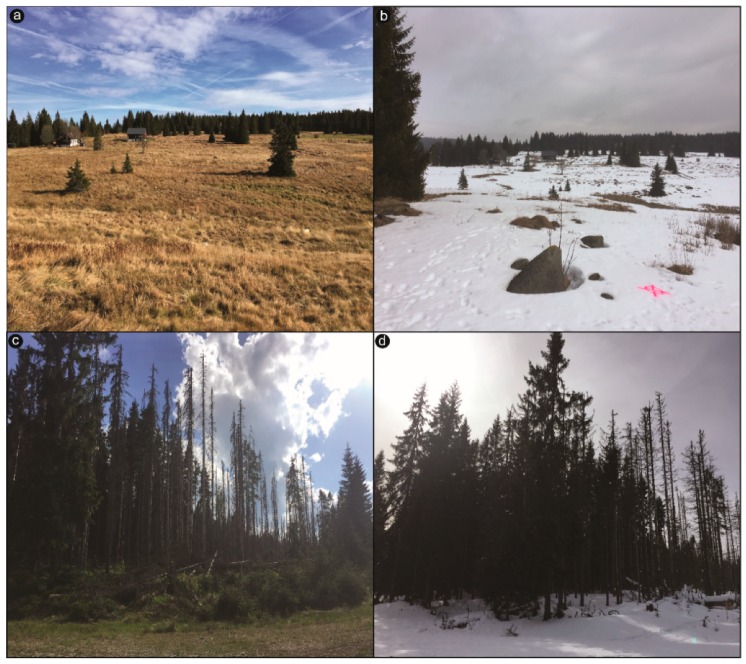
(**a**,**b**) Photographs of the meadow coverage with up to 0.40 m high grass and partly scattered rocks during summer and wintertime at the open area; (**c**,**d**) Dead wood lying on the ground during the snow-free period and at the base of the snow-cover.

Apparently, ground properties play a crucial role in the precise creation of accurate DSMs [5]. In case of the open field, meadow coverage (tall grass) at the base of the snow cover and scattered boulders had a strong influence on the results. There is a source of systematic error between H_GCP_ and H_UAV_ that can be assigned to the effect of vegetation, in particular to the grass cover. The height of the dense montane grass cover, which is in the study area dominated by Carex Rostrata and Nardus stricta L. [72], can reach up to 20–30 cm in the summer (Figure 12a). The grassland creates compact formations, covering the forest-free areas, where the optical imagery cannot reach the ground, which results in a corresponding shift of surface elevation. In winter, the grass is pressed down to the ground by the snow cover (Figure 12b), forming a snow-free layer of one centimeter below the snowpack. In the areas affected by forest disturbance, the lying trunks of dead trees and branches, the detection of the ground make a compact layer, which can result in an artificial shift of between the values, determined by geodetical surveying and the photogrammetric reconstruction. We assume that the snow-free DSM ground elevation is higher than the snow-covered DSM, causing the underestimation of H_UAV_ results, and overestimation of H_GCP_ and H_T_, as previously reported by [36,38,73]. This is also visible by the produced transects. Interpretation of the difference in snow depth profiles between the estimated H_UAV_ measurements and manual probing H_T_ is complicated especially in the forest zone by the fact that photogrammetric techniques do not work accurately above and below tree canopies resulting in zero snow depth values. Thus, extra care in interpretation needs to be taken in forests. Profiles of estimated H_UAV_ along the open area showed better correspondence with manual probing. This might be explained by the relatively homogenous terrain of a wide shallow slope characterized by meadow coverage. However, despite the lower or higher shifts of the estimated H_UAV_ measurements, we tried to minimize it by appropriate scheduling of the imaging campaign. The open field status was captured in early spring, just after the snowmelt (30 April 2017), before the start of the new vegetation season. The same applies to the forest site, which the dead wood on the ground could potentially cause error mapping during snow-free period and winter (Figure 12c,d), and also by difficulties applying UAV images above, below, and around trees, resulting in more significant gaps in the DSMs. Where available, the use of aerial LiDAR data as a reference dataset can significantly reduce this source of uncertainty and help to get a more accurate representation of the ground surface.

Vegetation effects are not the only issue. As the number of ground-based snow depth measurements is small, these data represent only a reduced variability concerning the complete range of variation of UAV-based snow depth evaluation. When estimating the snow depth for each pixel, the overall error derives from the two generated DSMs used for the subtraction. The error source on an SfM made DSM is divided into two categories: the photogrammetric reconstruction error; and, the georeferencing error. The photogrammetric error depends on the overall quality of the dataset, while the GNSS or post-processing quality cause the georeferencing error, the GCPs manual identification in the image, and the GNSS accuracy height point measurements [41]. This, in turn, depends on the quality of the sparse point cloud and, therefore, the quality of the methodology with which the images were acquired at the end [5]. For the future, an even better qualitative assessment of the snow-covered surface can be reached by applying the combined mapping of VIS and near-infrared (NIR) images, as has been done by [5,38]. Furthermore, a promising H_UAV_ approach might be to capture not only UAV images in the nadir position, but to combine them with oblique photos, taken by the same platform. The oblique imagery would enable minimizing the ground coverage by the tree crowns for the forest locations and to get more complex coverage of the ground. As the photogrammetric software, based on SfM technique allows a combination of the nadir and oblique imagery, such an approach could provide a better description of the ground surface below the tree stamps.

### 4.2. Validation of the Winter LAI

The assessment of the effect of different forest structures, forest disturbances, and open sites, on snow accumulation and snowmelt, is often done using modelling approaches (e.g., hydrological models, snowpack models, and land surface schemes of climate models) and collecting field data [8,39] based on measured or simulated field data. This means that these conceptualizations need input parameters to model real physical processes. Depending on the model, different predictors can be used to explain the variability of snow accumulation and ablation in distinct areas, as was done by [4]. They used the following predictors, i.e., elevation, slope, slope orientation, LAI, canopy openness, amount of shortwave radiation, irradiance, mean daily irradiance, snow depth, air temperature, and the date of sampling. To collect all these data is time intensive. Why shouldn’t it be used for the estimation of LAI_eff_, based on downward-looking images, where snow background is used instead of the sky fraction since UAV digital photogrammetry is a reliable tool to measure snow depth? Substituting this two-in-one approach for the heretofore standard methods helps resolve many of the aforementioned problems, although additional test data is still required to provide a reference standard. 

Although the LAI estimates tested using UAV produced, overall, sufficient LAI_eff_ values, even at different altitudes compared to the other devices, i.e., the LAI-2200 plant canopy analyzer, and DHP, small discrepancies between these methods appeared. Indeed, there was constant underestimation of the UAV-based LAI compared with the reference data. That might be the fact that snow was still present at few tree crowns during snow accumulation that caused these underestimations, even though the correspondence during snow ablation between UAV-based LAI_eff_ and LAI_eff_ LiCor-2200 or DHP_eff_ were slightly better due to the snow uncovered wood on the ground causing higher LAI_eff_ values. It has also been approved that there is an overestimation of the LAI_eff_ performed by LAI-2200 plant canopy analyzer compared to the other method results [44]. Here, a cap of 270° was used to hide both the sun and the operator from the sensor’s view and reduce the required clearing size for above-canopy readings in a denser forest. This cap is also preferable if the goal is to measure LAI_eff_ [57]. Nonetheless, a narrower view cap of 90° would probably be more appropriate, due to the diverse vegetation structure and the dynamic changes in spruce forest LAI in the study plot. Likewise, the LAI_eff_ values obtained by DHP present slightly overestimated than the LAI_eff_ UAV estimated. 

**Figure 13:**
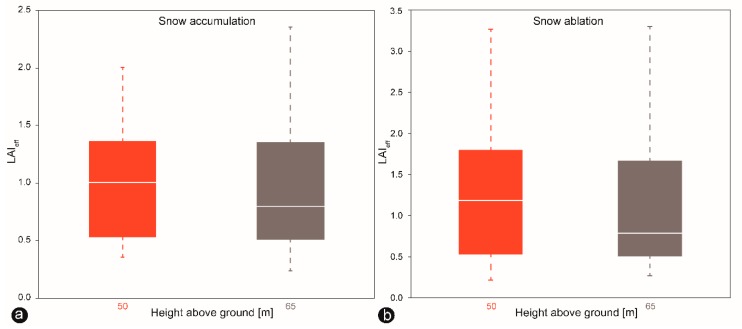
(**a**) Results of the UAV-based LAI_eff_ estimates. On the *y*-axes, the effective LAI is marked, and the flight altitude is plotted on the *x*-axis. The boxes identify the middle 50%, the median, and the maximum standard deviations for LAI points of UAV-based measurements at 50 m and 65 m altitude during snow accumulation. (**b**) Results of the UAV-based LAI_eff_ estimates at 50 m and 65 m altitude during snowmelt.

It is essential, therefore, to know from which altitude the LAI value should be taken, to compare with the ground measurements [61]. It was assumed that the optimal flight altitude for LAI_eff_ UAV estimates would be around 50 m. The difference in the results could have been caused by a different shooting zenith angle or camera zenith angle since the DHP and UAV methods had nearly similar software configurations, and, except for the different backgrounds, almost identical sampling methods in the experiment [74]. LAI extraction might be affected by the shooting angle regarding the proportion of leaf elements that might be influenced by the shooting angle of the different FOV of the cameras. To detect the same area with a smaller viewing angle (48°) on the UAV mounted camera, the observation height should be about tan (74°)/(48°) = 1.5 times the tree height, which varied in the test plot from 15 m to 20 m, with an average value of 17.5 m. Thus, the optimal flight altitude would be ~26 m. However, the tested flight heights at 50 m and 65 m show differences in capturing LAI (Figure 13). As the research area was covered by healthy and bark-beetle damaged trees, the LAI values were not kept at a very stable level. Variations in LAI_eff_ values appear mainly due to the heterogeneous structure of the forest. In the future, additional NIR or oblique imagery could be applied to improve the assessment quality of the DSM for snow depth mapping, as well as the LAI retrieval.

Moreover, in the case of UAV-based LAI estimates, the calculated LAI values might depend on the amount of snow cover because it also accounts the interception of snow-free dead lying branches and trees on the ground during snow ablation that the other methods are not taking into consideration. The ideal condition to perform UAV-based LAI retrieval is to have more snow on the ground because there will be fewer objects such as dead lying branches and trees interfere with the images, which could potentially affect the calculation of the LAI_eff_ values. However, this much is sure: that LAI decreased with increasing flight altitude during both snow accumulation and snowmelt, as observed in Figure 13 a,b. The lower the altitude is, the smaller is the area seen by the camera and the more significant is the variety of different scenes, whereas higher flights produce more averaged LAI values [61]. A similar trend was observed by [45,61,75].

### 4.3. Validation between Snow Depth and LAI

As expected, LAI_eff_ values negatively correlated to H_T_ data appeared at the investigated forest site more often during snow accumulation than during ablation (Figure 11). That was predicted by all methods used here. The lower the H_T_ measurements were, but the higher the LAI values, and vice versa. In general, increased LAI values mean more leaves in reality, leading to more surface covered by the canopy rather than by snow cover [44]. So, the presence of trees alters the processes that shape, in particular, snow cover accumulation. As a consequence, the amount of forest canopy, often described by estimates of LAI, are inversely related to snow accumulation [74]. However, a considerable variability between LAI_eff_ values and H_T_ were observed in measured comparisons between forest snow ablation and forest accumulation.

It is proven that the forest canopy interacts directly and indirectly with snow, and therefore impacts in significant ways on snow cover, especially during snowmelt periods [43]. The most direct is snow interception (e.g., canopy structure). Intercepted snow can be stored in the canopy, sublimated directly to the atmosphere when it is getting warmer [45,75], or change phase before reaching the underlying snow cover [76,77]. Since the investigated forest site consists of a paired forest and clearing structure, snow accumulates and melts differently. During snow accumulation, the clearing part contained the immense quantity of snow, where the LAI values were low. Whereas in the adjacent dense, healthy forest, less snow accumulated, due to increased interception and an increase in longwave radiation. 

With respect to snow melting, snow should last longer in the clearing part than in the denser forest. Concerning the low correlation between snow depth and LAI during snow ablation, and the tendency of some outliers showing a slight correlation of higher LAI values with higher snow depth. The reason for that might be that some of the intercepted snow sluffed off the canopy, particularly in areas of denser forest due to the higher LAI values, and was added to beneath-forest accumulation [12]. In fact, this assumption should be treated with care. It is clear that the snow variability cannot be determined via two parameters only, e.g., snow depth and LAI. There are more processes influencing snow distributions including the amount of shortwave radiation, air temperature, partial snowmelt periods, meltwater dripping from the canopy, wind sheltering, and snow redistribution due to the wind, elevation, slope orientation, and slope angle. Although the latter may not be relevant to a small forest area studied here, since it is a flat area, it might be suitable for open sites. The existing forest heterogeneity makes it difficult to postulate a clear direction of interpretation. It can be concluded that the use of LAI as a prediction parameter serves better for snow accumulation events than for snowmelt because it better describes snow interception. Hence, for snowmelt, factors that are more closely related to radiation and topography could give better results when describing the snow depth variability at a plot scale. Nevertheless, the substantial differences in spatial resolutions between manual and UAV-based snow depth measurements (meter vs. centimeters) are that small scale differences in both snow depth and vegetation structure cannot be captured by manual measurements either snow depth using probe or DHP. Therefore, to add more locations with manual measurements and consequent interpolation cannot bring the same level of detail associated with UAV which measures in cm resolution. 

## 5. Conclusions

This study has presented the potential of small footprint UAV data for deriving metrics such as snow depth (snow metric) and LAI (forest metric) using single flights. As was found in previous studies, we could show that it is possible to estimate snow depth and indirect LAI from UAV data by using RGB-based UAV imagery. Snow depth was computed by subtracting snow-free from snow-covered DSMs during different snow conditions and compared to fewer and with more manual ground measurements. Differences in measured values obtained by these snow samplings strategies draw distinctions. Effective winter LAI estimates conducted as well from downward-looking UAV images were compared to conventional optical methods (e.g., DHP and LAI-2200 canopy analyzer) producing reasonable but underestimated LAI_eff_ values. With increased flight height of the UAV, lower estimated LAI values were determined for all forest structures. 

The relation between LAI and snow depth was analyzed for different snow conditions. As expected, LAI_eff_ values negatively correlated to snow depth data during both snow conditions at the forest site. For snowmelt, the LAI as one of the predictors for snow distribution in forested areas represented weaker correlations. The reason is most likely that LAI is a much stronger predictor for snow accumulation, because it better describes the snow depth distribution during the accumulation period, while for snowmelt, more processes influence the snow variability. Elevation and topography have a marginal effect here, as the study plot in the forest zone is situated in a flat area. Thus, for snowmelt, factors that are more closely related to radiation and topography might give better results when describing the snow depth variability at a smaller scale. 

To this end, the results demonstrated that, with this combined method, snow depth and LAI retrieval could be recorded simultaneously with one UAV survey. The conjoint survey can significantly improve the efficiency of both aerial and field mapping of snow cover distribution in areas featuring variable structure and health status of the forest canopy. In such areas, it proved reliability in the determination of the snow depth variation along with the LAI, enabling the interpretation of the effects of canopy coverage on snow accumulation and ablation. 

However, the largest advantage of the UAV technology here is the high resolution and the high level of detail regarding the snow-covered terrain and the quick monitoring of indirect LAI compared to simple manual measurements of snow depth and point measurements of DHP or using LiCor-2200. By improving this method both parameters snow depth and indirect LAI might be used as input parameters for snow models captured in reduced time. 

Further work is required to examine the performance of snow depth change mapping in terms of the precision of snow depth change estimates under denser canopies where the non-vegetated surface is mainly covered and the use of infrared photography improves identification of different snow features such as windblown snow into openings in the forest, ice crusts, and wet snow surfaces. Attributes that need improvement as they affected the thresholding of binary images for the indirect LAI analysis were shadows and snow on tree crowns.

## Figures and Tables

**Table 1:** Details about the applied unmanned aerial vehicle (UAV) and camera system.

**UAV Details**	
UAV type	ARF-OktoXL
Dimension	73 × 73 × 36
Payload	2500 g
Gimbal	MK HiSight SLR2
Max altitude	100 m
Max distance	100 m
Flight time	Max. 45 min
Realistic flight time	15–28 min
Navigation	NaviCtrl V2.1 (IMU, barometer, GPS controller), MK-GNSS V4 GPS receiver (American GPS satellites, the European Galileo system, the Russian Glonass satellite or the Chinese BeiDou satellite system)
Wireless communication	Graupner MC-32 HoTT remote controller
LiPo battery	Vislero 5000, 14.8V 4S1P Flat
**Camera Details**	
Camera type	Lumix DMX-GX7
Sensor type	16MP Live MOS sensor
Sensor size (mm)	17.3 × 13.0 mm (in 4:3 aspect ratio)
Focal length	20 mm
Sensor resolution (MP)	16
ISO range	125–25,600
Weight (g)	402 (g)

**Table 2:** Input parameters of GLA. This setting was retained for both fisheye and orthographic image processing.

Input Parameter GLA	Value
Cloudiness index	0.5
Spectral fraction (0.25–25 μm)	1
Beam fraction	0.5
Clear-sky transmission coefficient	0.5
Solar constant (Wm^-2^)	1367

**Table 3:** Summary of the generated point clouds and the digital surface model (DSM) errors compared to the Global Navigation Satellite System (GNSS) elevation at ground control points (GCPs) and picked checkpoint (CP) locations of the open area and forest zone.

Flight Campaign	Date	Images	Flight Height [m]	Covered Area [km^2^]	Mean Ground Resolution [cm/pix]	Number of GCPs	Number of CPs	X Error RMSE [m]	Y Error RMSE [m]	Z Error RMSE [m]	XY Error RMSE [m]	Total RMSE [m]
Filipova Hut (open site; snow ablation)	24.3. 2017	963	62.2	0.056	1.16	8	6	0.041	0.039	0.055	0.056	0.079
Filipova Hut (snow-free)	30.4. 2017	1130	72.8	0.036	1.98	9	6	0.066	0.035	0.036	0.075	0.084
Ptaci brook (forest site; snow accumulation)	3.3. 2017	906	40.3	0.012	0.73	4	5	0.030	0.037	0.059	0.047	0.076
Ptaci brook (snow ablation)	15.3. 2017	1024	46	0.014	0.84	5	5	0.041	0.050	0.026	0.065	0.067
Ptaci brook (snow-free)	30.4. 2017	564	60.7	0.015	1.12	5	4	0.046	0.030	0.056	0.055	0.078

**Table 4:** Statistics of the evaluation between estimated (H_UAV_) snow depths and ground measurements at both study sites. The subtracted H_GCP_ snow depth values from the estimated H_UAV_ measurements are based on one pixel. On the contrary, the subtracted H_T_ samples from the estimated H_UAV_ are based on the mean value within 1 m radius.

**H_GCP_-H_UAV_** **(One Pixel-Base; z values)**	**Open Area** **(Snow Ablation)** **n = 14**	**Forest** **(Snow Accumulation)** **n = 9**	**Forest** **(Snow Ablation)** **n = 10**
Mean bias [m]	0.22	0.21	0.29
SD [m]	0.11	0.19	0.19
MAE [m]	0.09	0.16	0.13
RMSE [m]	0.08	0.15	0.09
**H_T_-H_UAV_** **(Mean Value of 1 m Radius; z values)**	**Open Area** **(Snow Ablation)** **n = 105**	**Forest** **(Snow Accumulation)** **n = 36**	**Forest** **(Snow Ablation)** **n = 36**
Mean bias [m]	0.08	0.14	0.14
SD [m]	0.14	0.29	0.27
MAE [m]	0.19	0.24	0.22
RMSE [m]	0.16	0.32	0.31
